# Protective Effects of Notoginsenoside R1 via Regulation of the PI3K-Akt-mTOR/JNK Pathway in Neonatal Cerebral Hypoxic–Ischemic Brain Injury

**DOI:** 10.1007/s11064-018-2538-3

**Published:** 2018-04-25

**Authors:** Liu Tu, Yan Wang, Di Chen, Ping Xiang, Jingjing Shen, Yingbo Li, Shali Wang

**Affiliations:** 1 Cerebrovascular Diseases Laboratory, Institute of Neuroscience, Chongqing Medical University, No. 1, Yixueyuan Road, Yuzhong District, Chongqing, 400016 China; 2 Department of Cardiology, Children’s Hospital of Chongqing Medical University, Chongqing, China

**Keywords:** Notoginsenoside R1, Hypoxic-ischemic brain damage, PI3K-Akt-mTOR/JNK signaling pathway, Apoptosis, Estrogen receptors

## Abstract

Notoginsenoside R1 (NGR1) is a predominant phytoestrogen extracted from *Panax notoginseng* that has recently been reported to play important roles in the treatment of cardiac dysfunction, diabetic kidney disease, and acute liver failure. Studies have suggested that NGR1 may be a viable treatment of hypoxic-ischemic brain damage (HIBD) in neonates by reducing endoplasmic reticulum stress via estrogen receptors (ERs). However, whether NGR1 has other neuroprotective mechanisms or long-term neuroprotective effects is unclear. In this study, oxygen-glucose deprivation/reoxygenation (OGD/R) in primary cortical neurons and unilateral ligation of the common carotid artery (CCL) in 7-day-old postnatal Sprague Dawley (SD) rats followed by exposure to a hypoxic environment were used to mimic an HIBD episode. We assessed the efficacy of NGR1 by measuring neuronal damage with MTT assay and assessed brain injury by TTC staining and brain water content detection 24–48 h after OGD/HIE. Simultaneously, we measured the long-term neurophysiological effects using the beam walking test (5 weeks after HI) and Morris water maze test 5–6 weeks after HI. Expression of PI3K-Akt-mTOR/JNK (24 h after HI or OGD/R) proteins was detected by Western blotting after stimulation with HI, NGR1, LY294002 (PI3K inhibitor), 740Y-P (PI3K agonist), or ICI 182780(estrogen receptors inhibitor). The results indicated that NGR1 exerted neuroprotective effects by inhibiting neuronal apoptosis and promoting cell survival via the PI3K-Akt-mTOR/JNK signaling pathways by targeting ER in neonatal hypoxic–ischemic injury.

## Introduction

Hypoxic-ischemic brain damage (HIBD) in neonates is an important risk factor for many severe human neurological dysfunctions, such as motor and learning disabilities, cerebral palsy, epilepsy, and even death [1–3]. In spite of the major advances in modern medical technology and the increased understanding of fetal and neonatal pathologies, neonatal hypoxic–ischemic encephalopathy (HIE) is still an unresolved serious condition that leads to significant mortality and long-term morbidity [4–7]. Presently, there are no well-established effective therapies for neonatal HIE [8]. Hypoxic–ischemic brain injury directly results in a large amount of neuronal death. Research suggested that an important way causing neuronal loss was apoptosis, especially in the penumbra area [9]. Malagelada et al. [10] found that there were at least 50% of dying cells which performed morphological characteristics of apoptosis in OGD-treated cortical neuron cultures. Therefore, enhancing neuronal survival, reducing apoptosis have become the most important strategies for solving neurological diseases [11].

Notoginsenoside R1 (NGR1) is a predominant phytoestrogen extracted from *P. notoginseng*. NGR1 was recently reported to possess anti-inflammatory, antioxidant, and anti-apoptotic properties, and may play important roles in the treatment of cardiac dysfunction [12–15], acute liver failure [16], and diabetic kidney disease [17]. Meng et al. [18] found that 3-day pretreatment with NGR1 significantly reduced cerebral infarct volume in an adult rat model, while pretreatment with NGR1 for 24 h prevented apoptosis induced by oxygen glucose deprivation/reoxygenation (OGD/R) in primary cortical neurons. Our past study [19] indicated that NGR1 treatment exerted neuroprotective effects in the acute phase of a neonatal HIBD model. It is worth noting that neonatal HIBD often leads to long-lasting neurological deficits such as mental deficiency, cerebral palsy, and learning disabilities, which develop in the immature brain. These consequences have seriously affected the quality of life of children with HIE. Whether NGR1 treatment can promote the long-term recovery of neurological function after HIBD has not yet been reported and is worth exploring.

Research [13, 15, 18, 19] has indicated that NGR1 may perform its functions through estrogen receptors (ERs). The classic ERs have two major subunits, estrogen receptor α (ERα) and estrogen receptor β (ERβ). Within the brain, ERα/β are found in cognitive brain regions associated with learning and memory, such as the cerebral cortex, hippocampus, and basal forebrain [20, 21]. A number of studies have shown that ERs play an important role in organ ischemic injury. Liu et al. [22] found that calycosin exhibited an anti-apoptotic effect via ERα/β and improved Akt phosphorylation in cardiomyocytes. Hsu et al. [23] suggested that 17β-estradiol (E2) treatment reversed hepatic injury following hemorrhagic shock and resuscitation through ERs-related p38 MAPK-dependent HO-1 upregulation. Wang et al. [24] reported that E2 offered protection against retinal ischemic injury by inducing upregulation of SDF-1 expression through activation of ERs. Activating ERs were found to provide protection for CA1 neurons in ischemic injury, while ICI 182780 (the broad-spectrum ERs antagonist) abolished the protection [25].

As an important signal transduction pathway, PI3K-Akt-mTOR/JNK is involved in many cellular processes, including cell apoptosis, survival and proliferation [26, 27]. Phosphatidylinositol 3 kinase (PI3K) is an intracellular phosphatidylinositol kinase which consists of a catalytic subunit (p110) and a regulatory subunit (p85) [28, 29]. Protein kinase B (Akt), a serine/threonine kinase, is a primary downstream target in the transduction pathway of PI3K signaling. Akt is a key information molecule that promotes cell survival, inhibits apoptosis [30] and maintains normal functions [31]. Activated Akt can transmit signals to a variety of downstream substrates. The common downstream proteins include TSC1/2-Rheb-mTOR [32], pro-apoptotic factor JNK, NFκb, and frontal transcription factor FKHR [33]. Mammalian target of rapamycin (mTOR) is a serine/threonine kinase that can benefit cell growth, survival, and metabolism [32]. The main targets of activated mTOR are ribosomal protein S6 kinase (p70S6K) and eukaryotic initiation factor 4E binding protein 1 (4E-BP1). Among them, p70S6K is mainly involved in cell-cycle regulation and contributes critically to cell survival. Activated p70S6K promotes the synthesis of ribosome translation regulator protein, resulting in the positive regulation of protein synthesis. Through the phosphorylation of 4E-BP1, mTOR regulates cap-dependent protein translation and promotes the proliferation of neurons. JNK which also can be regulated by Akt directly or indirectly controls a number of transcriptional and non-transcriptional processes, including inflammation and cell death or survival [26, 34–39].

Many studies have shown that PI3K-Akt-mTOR/JNK signaling plays a major role in cerebral hypoxic–ischemic injury [26, 32, 40, 41]. Some researchers [42–44] have found that Akt signaling, which is activated after transient cerebral ischemia, inhibits delayed neuronal apoptosis and promotes cell survival. Activation of the mTOR pathway is sufficient for promoting both neuron survival and axon regeneration [45, 46]. Research [26, 47, 48] indicates that the JNK pathway is also involved in ischemia-induced neuronal apoptosis. Hence, a number of researchers have proposed that JNK may be a target for the treatment of neuronal necrosis and that the inhibition of the JNK signaling pathway may reduce the apoptosis caused by ischemic brain damage [49–51].

Some studies have reported that NGR1 could protect the heart from septic shock via the activation of ERα and PI3K/Akt signaling [13]. NGR1 activated Nrf2/ARE signaling and upregulated phase II antioxidant enzymes in PC12 cells via ERs [52]. Our previous findings suggested that NGR1 could inhibit endoplasmic reticulum stress-induced neuronal apoptosis and brain damage via ERs [19]. However, it remained unclear whether NGR1 could exert neuroprotective effects and reduce neuron apoptosis via ERs by acting on the PI3K-Akt-mTOR/JNK signal pathway in a neonatal hypoxic–ischemic brain damage (HIBD) model.

In this study, we investigated the neuroprotective effects of NGR1 in a neonatal HIBD model, especially concerned whether NGR1 had a contribution to the long-term recovery of neurological function in the HIE. Furthermore, we explored the neuroprotective mechanisms of NGR1 by inhibiting neuronal apoptosis and promoting cell survival via the ERs and PI3K-Akt-mTOR/JNK signaling pathway.

## Materials and Methods

### Drug Preparation

NGR1 (chemical structure C_47_H_80_O_18_, molecular weight = 933.13, purity > 98%) was from Sigma-Aldrich (Sigma-Aldrich, St. Louis, MO). ICI-182780 (an estrogen receptor inhibitor), LY294002 (an inhibitor of PI3K) and 740Y-P (an agonist of PI3K) were from Tocris (London, UK), Selleck Chemicals (Houston, Texas, USA),and Selleck Chemicals (Houston, Texas, USA), respectively.

### Animals

Seven-day-old Sprague–Dawley (SD) male rats and rat fetuses (18 days) were provided by the Animal Department of Chongqing Medical University (Chongqing, China). All experiments were put into practice in accordance with the National Institutes of Health Guide for the Care and Use of Laboratory Animals. All protocols were ratified by the Animal Ethics Committee of Chongqing Medical University and efforts were made to reduce animal suffering.

### Cell Culture and Drug Treatment

The experiment was conducted according to previously described methods [19, 53]. Dissociated cultures of cortical neurons were harvested from time-mated embryonic day 18 (E18) rat brains using established protocols. Cerebral cortices were excised and hatched in Ca^2+^- and Mg^2+^-free HBSS solution. The tissues were mechanically separated and then digested in 0.25% trypsin (with 0.02% EDTA) for 7 min at 37 °C. After trypsinization was terminated, the digests were centrifuged for 5 min at 1000 rpm. The centrifuged cells were resuspended in Neurobasal medium (Gibco, Gaithersburg, MD) with 2% B-27 supplement (Gibco) and 2 mmol/l l-glutamine (Invitrogen, Gaithersburg, MD). Cells were subcultured in 96-well plates (5 × 10^4^ cells/well) for 3-(4,5-dimethyl-2-thiazolyl)-2,5-diphenyl-2H-tetrazolium bromide (MTT) assays, in 24-well plates (1 × 10^5^ cells/well) for lactate dehydrogenase (LDH) determination and in 6-well plates (1 × 10^6^ cells/well) for other experiments. Plates were precoated with polyethylenimine (0.05 mg/ml, Sigma-Aldrich) overnight at 37 °C. Cultures were maintained in a Heraeus CO_2_ incubator (Thermo Fisher Scientific, Rockford, IL) containing 5% CO_2_ and 95% air at 37 °C. Cultures were used for experiments on the fifth day in vitro. Cells were treated with NGR1 (10 µmol/l) [19] when subjected to oxygen glucose deprivation and reoxygenated. ICI 182780 (0.1 µmol/l) [19] was used to preprocess cells 2 h before OGD. LY294002 (20 µmol/l) and 740Y-P (20 µmol/l) were applied to cells 1 h before OGD. ICI 182780, LY294002, and 740Y-P were dissolved in dimethyl sulfoxide (DMSO). DMSO acted as a vehicle with a concentration of 1%.

### Oxygen Glucose Deprivation/Reoxygenation

OGD/R was accomplished using day-5 cultured primary cortical neurons to imitate cerebral ischemic/reperfusion injury. OGD/R was achieved using a modification of a previously described procedure [19]. After the cells were washed once with phosphate-buffered saline (PBS), culture plates were replenished with glucose-free Dulbecco’s Modified Eagle’s Medium. Cultures were placed in an anaerobic chamber (Thermo Fisher Scientific) and incubated in an anaerobic gas mixture (1% O_2_, 5% CO_2_, and 94% N_2_) at 37 °C. After 1.5 h, cultures were returned to a normoxic environment from the anaerobic chamber. Simultaneously, the culture plates were refilled with Neurobasal medium, and cultures were allowed to reoxygenate for 4–24 h.

### Hypoxic-Ischemic Brain Damage Model

HI was imitated by unilateral ligation of the common carotid artery (CCL) followed by 2.5 h of hypoxia in 7-day-old SD rats. Rat pups were anesthetized with isoflurane (2.5%) and supine fixed in the thermostat console. A longitudinal midline incision disinfected by iodophor disinfectant was made in the anterior neck. After the right common carotid artery was identified and freed from the surrounding tissues, without any damage to the right vagus nerve, it was double ligated and transected between the ligatures. The pups were then returned to a heating pad for 1 h for recovery. Simultaneously, an airtight chamber containing 7% humidified oxygen and 93% N_2_ was prepared using a heating pad to maintain the temperature at 35–39 °C. Then the HI animals were placed in the chamber for 2.5 h. Sham animals received an incision but did not undergo CCL treatment, and the pups were placed in a similar container but not exposed to a hypoxic environment. After modeling, all pups were returned to their dams. NGR1 (15 mg/kg q 12 h, for 2 days) [19] was administered to the pups by intraperitoneal injection after CCL immediately, before exposure to the hypoxic environment. ICI-182780 (2 mg/kg) was administered to pups 2 h before CCL treatment by intraperitoneal injection [19].

### Cell Viability Assessment

An MTT assay was used to test cell viability. Four or 24 h after the OGD/R injury, cells were incubated with MTT (0.05 mg/l) for 4 h at 37 °C. The culture medium was then completely removed, and all wells were filled with 100 µl DMSO to dissolve the formazan crystals. Absorbance was surveyed at 570 nm using a microplate reader (Bio-Rad Model 680, Bio-Rad, Hercules, CA). Cell viability was calculated using the formula (mean experimental absorbance/mean control absorbance) × 100%.

### Measurement of Cell Membrane Integrity

The rate of LDH release was used to estimate the membrane integrity of cells. The supernatant of each well was collected, and the LDH content was determined using an LDH assay kit according to the manufacturer’s instructions (Nanjing Institute of Jiancheng Biologic Engineering, Nanjing, China). For the positive control, the supernatant of the cells was collected after cells were lysed using 0.25% Triton X-100. The level of LDH release was calculated using the formula (experimental LDH activity/positive control LDH activity) × 100%.

### Morris Water Maze

Neurocognitive outcomes were measured by using the Morris water maze (WM) test with a computerized video tracking system (BW-mwm101, Shanghai BioWill Co., Ltd., China) 5–6 weeks after modeling. The WM consisted of a circular pool 120 cm in diameter and 47 cm in height, containing water 30 cm deep. A hidden submerged platform (9 cm diameter) was placed in the second quadrant 2.5 cm below the water surface for rats to step on and escape from the water. Rats could identify the position of the platform using visual clues placed on the walls. The time to locate the submerged platform (defined as the latency, with cutoff time 60 s) was measured. Every day, each rat performed four trials starting from different quadrants. The test lasted for 5 days. On testing day 6, each rat performed a probe trial (60 s cutoff) without a platform. All of the activities were video recorded, and the animals’ swimming paths were measured for quantification of time, frequency, and latency [54, 55] using the ANY-maze Animal Behavioral Video Analysis System (Shanghai Bio-will Co., Ltd, China).

### Beam Walking Test

Coordination and integration of motor movement was assessed with a beam (80 cm × 2.0 cm × 2.5 cm; 60 cm above floor) walking test 5 weeks after modeling. Each rat was tested 3 times, for 2 min each time. The ratio scale was modified from Ohlsson [56] and Feeney [57]. Balance performance on the beam was graded as follows: 0, the rat falls down and cannot walk on the beam; 1, the rat is unable to walk on the beam but can sit on the beam; 2, the rat falls down while walking; 3, the rat can traverse the beam, but the affected hind limb does not aid in forward locomotion; 4, the rat crosses the beam with more than 50% foot slips; 5, the rat traverses the beam with fewer than 50% foot slips; 6, the rat successfully crosses the beam with no foot slips.

### Evaluation of Brain Damage 6 Weeks After Modeling

Hemispheric weight loss has been used as an important variable for assessing brain atrophy in neonatal HI model [58]. After Morris water maze test, the brains were extracted and the hemispheres were cut along the center line and weighed on a high-precision balance. The brain weight ratio (%) was calculated using the formula (weight of ipsilateral hemisphere/weight of contralateral hemisphere) × 100%.

### Evaluation of Infarction Volume

2,3,4-tiphenyl tetrazolium chloride (TTC) (Sigma-Aldrich, MO) staining is a reliable way to evaluate infarction volume. Using this method, the brain sections were prepared as follows: First, the brains were removed and frozen at − 20 °C for 10 min. Next, consecutive 2 mm coronal sections were obtained by slicing the brains with Brain Matrix (ASI Instruments, Warren, MI). The subsequent incubation of the sections was performed in a dark environment with 25-min immersion in 2% TTC solution at 37 °C. Finally, the sections were immersed in a 4% formaldehyde solution. TTC stained normal areas of brain deep red but did not stain infarcted tissue. Infarction volumes were measured and analyzed with ImageJ software (NIH Image, Version 1.61, Bethesda, MD, USA) as described previously [19].

### Brain Water Content Detection

Rats were sacrificed 24 h after HI for brain water content measurement. The wet weight of the brain sample was measured immediately after harvest. The brain was then placed in an oven at 105 °C for 24 h and weighed again to determine the dry weight [59]. Brain water content (%) was calculated using the formula[(wet weight − dry weight)/wet weight] × 100%.

### TUNEL Staining

Coronal brain slices were stained with neuron-specific nuclear protein (NeuN) and terminal deoxynucleotidyl transferase-mediated nick-end labeling (TUNEL) to measure apoptotic neurons 24 h after HI. After dewaxing by xylene, sections were subjected to gradient hydration. The slices were incubated with anti-NeuN (1:50, Abcam) and Alexa Fluor 555-labeled goat anti-mouse IgG (1:100, Beyotime Institute of Biotechnology). Afterward, samples were added to the TUNEL reaction mixture (Thermo Fisher Scientific) for an incubation time of 60 min at 37 °C in a humidified atmosphere in the dark. Then, DAPI was used to incubate the samples for 2 min. Apoptotic cells were photographed under a microscope (Olympus) with an excitation wavelength of 450–500 nm (green) and a detection wavelength of 515–565 nm (red). Three coronal brain sections were selected from each brain (six animals in each group), and the numbers of positive cells (neurons) in the ipsilateral cerebral cortex was counted for each section at high magnification in five visual fields. The proportion of TUNEL-positive cell nuclei was determined by dividing the number of TUNEL-positive nuclei by the number of total nuclei.

### Western Blots

Protein expression was evaluated through Western blot analysis. Cells or brain tissues (Respectively taking the contralateral hemisphere and ipsilateral hemisphere) were homogenized by lysis buffer (Beyotime Institute of Biotechnology). The insoluble material was removed by centrifugation at 12,500 rpm for 15 min at 4 °C. The supernatants of the lysate were collected to measure the protein concentration with a BCA Protein Assay Kit (Thermo Fisher Scientific). Protein samples were denatured for 5 min at 100 °C after being mixed with sodium SDS gel-loading buffer. Then, samples were separated by SDS–polyacrylamide gel electrophoresis and transferred to a polyvinylidene membrane (the specific conditions of electrophoresis and transfer varied according to the molecular weight of the target protein). Membranes were blocked for 2 h in 5% nonfat dry milk in Tween/Tris-buffered saline (TTBS) at room temperature. The membranes were then incubated with the primary antibody. After incubation overnight at 4 °C, the membranes were washed with Tris-buffered saline and incubated with a secondary antibody for about 2 h at room temperature. Bands were scanned and densitometrically analyzed by automated ImageJ software (NIHImage, Version 1.61).

### Statistical Analysis

All data are expressed as mean ± SEM statistical analyses were carried out by SPSS version 17.0 (SPSS, Chicago, IL). One-way analysis of variance was used to evaluate the significance of differences among experimental groups. A *p* value of 0.05 was regarded as the level of statistical significance.

## Results

### NGR1 Attenuated OGD/R-Induced Cortical Neuron Damage Mediated by Estrogen Receptors

**Fig. 1:**
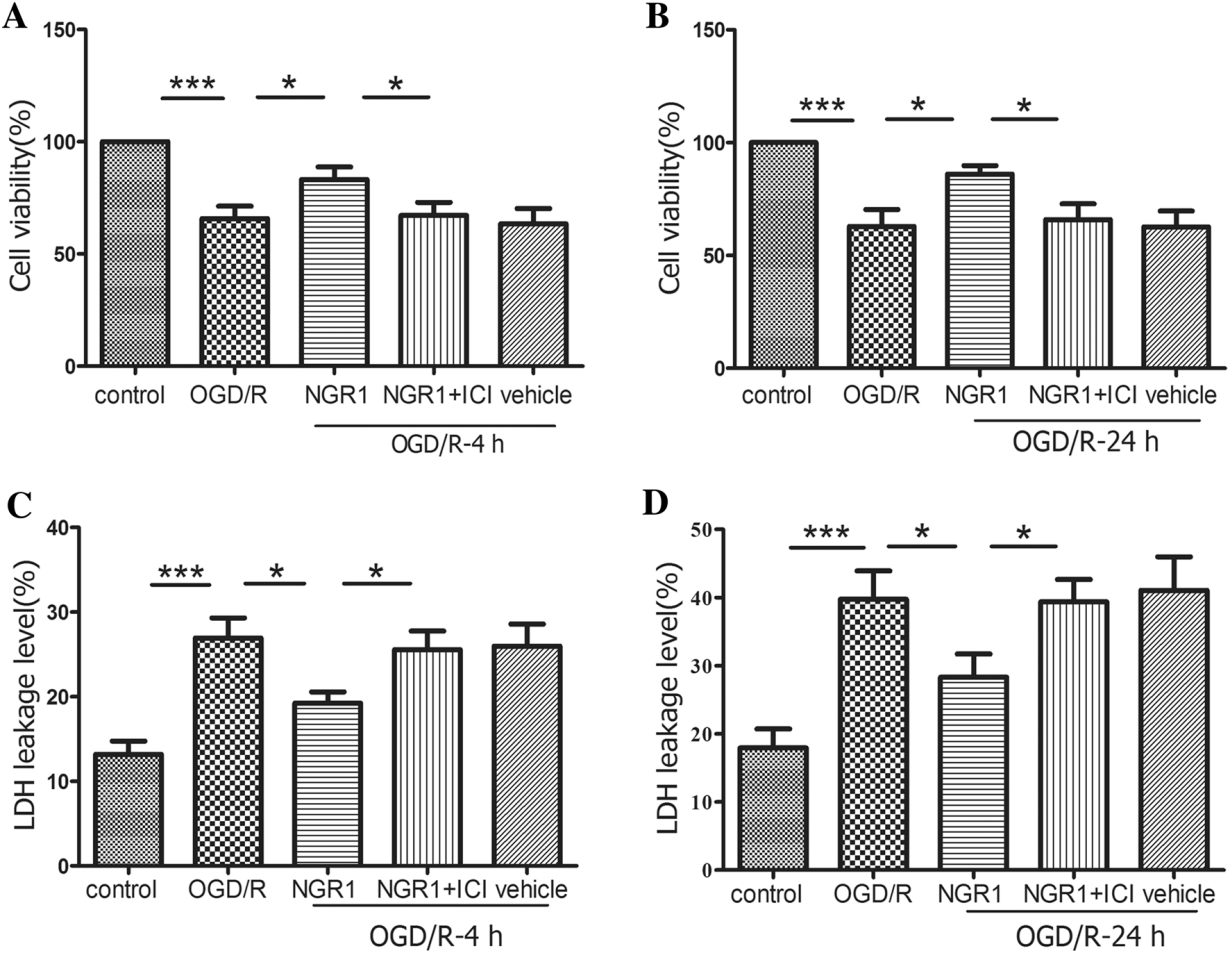
The effects of NGR1 treatment on neuron injury after OGD/R via estrogen receptors. **a** and **b** At 4 and 24 h after OGD/R, NGR1 increased cell viability compared with the OGD/R group, ICI 182780 pretreatment could abolish this effects. The OGD/R + NGR1 + ICI 182780 group had lower cell viability compared with the OGD/R + NGR1 group. **c** and **d** At 4 and 24 h after OGD/R, NGR1 treatment reduced LDH release in neurons and ICI 182780 reversed this effects. Data are expressed as the mean ± SEM for n = 6. *p < 0.05; **p < 0.01; ***p < 0. 001

As the main component of the phytoestrogen from *P. notoginseng*, NGR1 protected the cortical neurons from injury induced by OGD/R, but this effect could be blocked by ERs blocker ICI 182780. Neuronal damage was measured by MTT assay and LDH leakage performed at 4 or 24 h after OGD/R (Fig. 1). The results showed that NGR1 (10 µmol/l) significantly improved neuronal cell viability (83.17 ± 13.68 vs. 65.71 ± 13.60%, *p* < 0.05, at 4 h after OGD/R; 86.01 ± 9.17 vs. 62.85 ± 18.31%, *p* < 0.05, at 24 h after OGD/R) and reduced the LDH leakage rate (19.23 ± 3.24 vs. 26.92 ± 5.86%, *p* < 0.05, at 4 h after OGD/R; 28.31 ± 8.34 vs. 39.75 ± 10.20%, *p* < 0.05, at 24 h after OGD/R) in the cortical neuron OGD/R model compared with the OGD/R group.

However, ICI 182780 could suppress these neuroprotective effects of NGR1. In the OGD/R + NGR1 + ICI 182780 group, the cell viability was significantly reduced (67.19 ± 14.28 vs. 83.17 ± 13.68%, *p* < 0.05, at 4 h after OGD/R; 65.81 ± 17.36 vs. 86.01 ± 9.17%, *p* < 0.05, at 24 h after OGD/R), and the LDH leakage rate was significantly increased (25.18 ± 4.76 vs. 19.23 ± 3.24%, *p* < 0.05, at 4 h after OGD/R; 39.36 ± 8.02 vs. 28.31 ± 8.34%, *p* < 0.05, at 24 h after OGD/R) compared with the OGD/R + NGR1 group. There was no significant difference in cell viability or LDH leakage rate between the DMSO vehicle group and the OGD/R group.

### NGR1 Attenuated HI-Induced Brain Injury in Newborn Rats Mediated by Estrogen Receptors

**Fig. 2:**
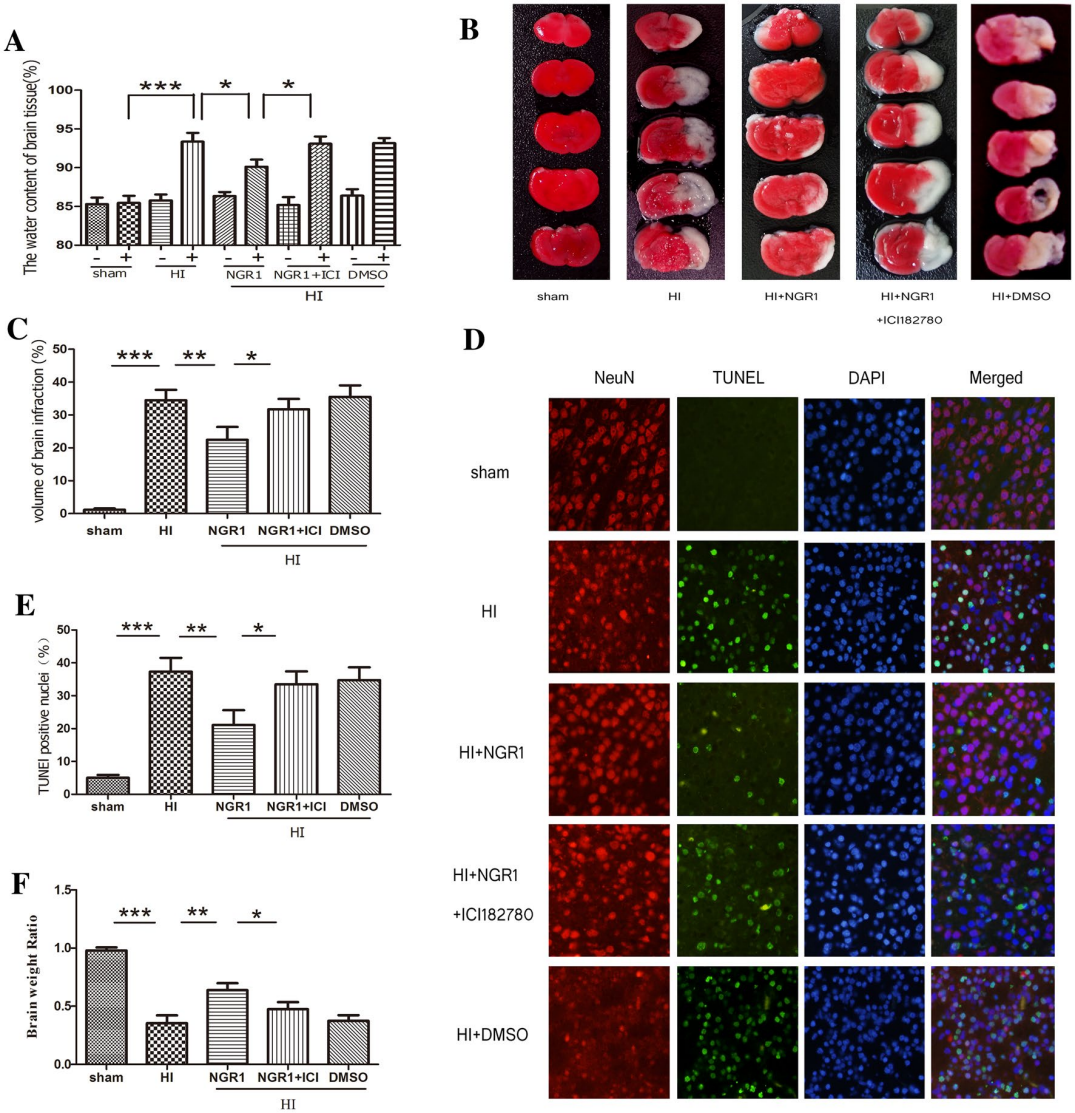
The effects of NGR1 on brain injury after HI via estrogen receptors. **a** The water content in the ipsilateral hemisphere was significantly decreased in the NGR1 treatment group compared with the HI group. There was also a significant increase in water content in the HI + NGR1 + ICI 182780 group compared with the HI + NGR1 group. (sham n = 7, HI n = 9, NGR1 n = 9, HI + NGR1 + ICI 182780 n = 8, HI + DMSO n = 7; + means ipsilateral, − means contralateral). **b** and **c** NGR1 could reduce the infarction area, but the neuroprotective effect was blocked by ICI 182780. The HI + NGR1 + ICI 182780 group showed a larger infarction area than the NGR1 treatment group (sham n = 6, HI n = 9, NGR1 n = 9, HI + NGR1 + ICI 182780 n = 8, HI + DMSO n = 7). **d** and **e** The number of TUNEL-positive cortical neurons were greater in the HI group than in the HI + NGR1 group, but the administration of ICI 182780 could inhibit the protective effect of NGR1. A large number of TUNEL-positive cortical neurons were also found in the HI + NGR1 + ICI 182780 group (n = 6). Data are expressed as mean ± SEM. **f** The ipsilateral hemisphere weight was significantly decreased in the HI group compared with the NGR1 treatment group 6 weeks after HI. ICI 182780 could block this effect. There was also a significant reduction of ipsilateral hemisphere weight in the HI + NGR1 + ICI 182780 group compared with the HI + NGR1 group (sham n = 8, HI n = 9, HI + NGR1 n = 9, HI + NGR1 + ICI 182780 n = 9, HI + DMSO n = 9). *p < 0.05; **p < 0.01; ***p < 0.001

Brain edema was detected at 24 h after HI (Fig. 2a), as indicated by increased brain water content. Compared with the sham group (85.46 ± 2.43%), the ipsilateral hemisphere water content was significantly increased in the HI group (93.36 ± 3.41%, *p* < 0.001 vs. the sham group). The ipsilateral hemisphere water content was significantly reduced by treatment with NGR1 (90.12 ± 2.78%, *p* < 0.05 vs. the HI group), but this effect could be reversed by ICI 182780 (93.09 ± 2.63%, *p* < 0.05 vs. the HI + NGR1 group).

Infarct volume was used to evaluate brain damage at 48 h after HI injury. As shown in Fig. 2b, c, HI caused an increased magnitude of infarction in the right hemisphere (34.49 ± 9.49%), and the infarct volume was significantly reduced in the HI + NGR1 group (22.49 ± 11.63%, *p* < 0.01 vs. the HI group). The result supported the neuroprotective effect of NGR1. Quantitative comparisons of the infarct volumes of the HI + NGR1 group and the HI + NGR1 + ICI 182780 group showed that the degree of infarction was intensified in the latter (31.74 ± 8.90%, *p* < 0.05 vs. the HI + NGR1 group).

The cortical neuronal apoptosis was observed at 24 h after HI injury. Few TUNEL-positive cortical neurons were found in the sham group, while in the HI group, neuronal apoptosis was 37.35 ± 10.16%. In comparison, neuronal apoptosis was 21.10 ± 11.00% in the HI + NGR1 group (*p* < 0.01 vs. the HI group), however the neuroprotective effect of NGR1 could be reversed by ICI 182780 (33.48 ± 9.53%, *p* < 0.05 vs. the HI + NGR1 group) (Fig. 2d, e).

In order to observe the long-term effect of NGR1 on HIBD, the hemisphere weight was estimated at 6 weeks after surgery [38]. The HI injury caused severely brain atrophy, marked by a decrease in the right-to-left hemispheric weight ratio in HI group(0.35 ± 0.20, *p* < 0.001 vs. the sham group), but the brain atrophy was significantly improved in the HI + NGR1 group (0.64 ± 0.18, *p* < 0.01 vs. the HI group) (Fig. 2f). Blockage of ERs reversed the neuroprotective effect (0.48 ± 0.19, *p* < 0.05 vs. the HI + NGR1 group).

### NGR1 improved neurobehavioral function Mediated by Estrogen Receptors

**Fig. 3:**
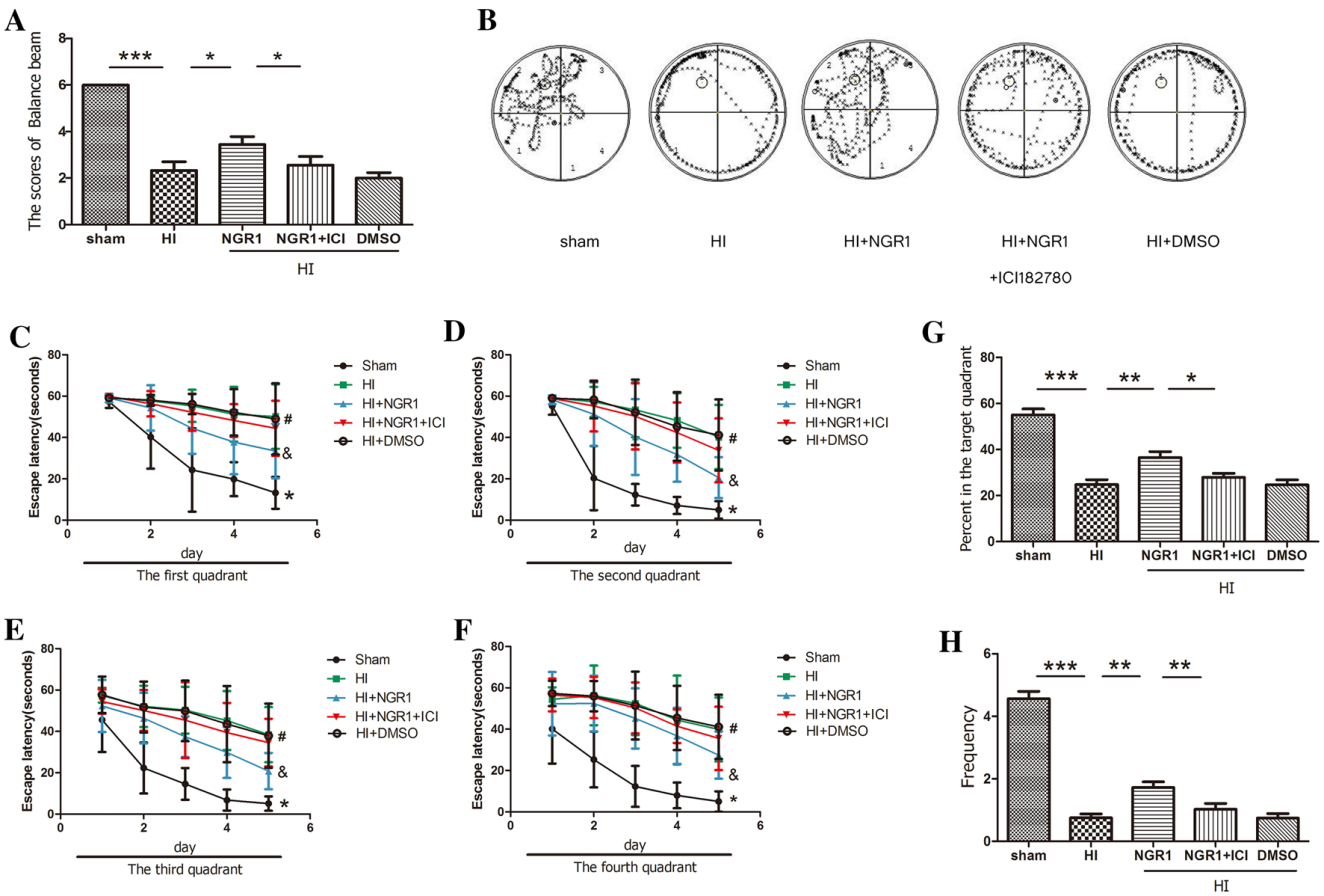
Neurobehavioral effects of NGR1 5–6 weeks after HI via estrogen receptors. **a** Balance performance was severely impaired in the HI group at 5 weeks after HI, but NGR1 treatment significantly improved balance performance. The protective effect of NGR1 was blocked by ICI 182780. **b**–**h** The Morris water maze test was performed 5–6 weeks after HI. The results showed that the latencies of the HI group were significantly higher than those of the sham group (*HI group vs. sham group *p* < 0.05, #HI group vs.HI + NGR1 group *p* < 0.05, &HI + NGR1 group vs. HI + NGR1 + ICI 182780 group p < 0.05) (**b**–**f**). The percentage of time spent in the target quadrant **g** and the frequency of crossing the target platform **h** were significantly higher in the sham group than those in the HI group; NGR1 treatment could increased the percentage of time and the frequency compared to the HI group. However, the protective effects could be reversed by ICI 182780 (**b, g**–**h**). Data are expressed as mean ± SEM. Sham n = 8, HI n = 9, HI + NGR1 n = 9, HI + NGR1 + ICI 182780 n = 9, HI + DMSO n = 9. *p < 0.05; **p < 0.01; ***p < 0.001

Balance performance was severely impaired in the HI group at 5 weeks after HI insult (Fig. 3a). In contrast, rats treated with NGR1 showed significantly improved balance performance compared with the HI group (3.44 ± 1.01 vs. 2.33 ± 1.12, *p* < 0.05). However, the protective effect of NGR1 was blocked by ICI 182780. The result showed significantly reduced scores in the HI + NGR1 + ICI 182780 group (2.56 ± 1.13, *p* < 0.05 vs. the HI + NGR1 group).

NGR1 could improve spatial learning and memory function recovery, as indicated by the Morris water maze test which was detected 5–6 weeks after neonatal HI injury. The rats’ escape latency reflected their spatial learning and memory impairments. The results (Fig. 3b–f) showed that the latencies of the sham group were significantly shortened after 2 days of training, which indicated that the sham group rats had intact learning and memory capacities. At the end of the fifth day of training, almost all rats could aim to move in the direction of the platform. After the platform was removed, some sham group rats went directly to the location of the platform and wandered nearby, which suggested that the rats had remembered the location of the platform. However, the HI group rats mostly swam in the pool without showing obvious signs of proximity to the platform. The latencies of the HI group in each of the four quadrants were 50.11 ± 15.19, 40.23 ± 15.53, 38.43 ± 13.32, 39.89 ± 15.46 s, respectively. They were higher than those of the sham group (13.21 ± 7.70, 4.98 ± 4.20, 5.12 ± 3.46, and 5.01 ± 4.88 s, respectively; *p* < 0.05 vs. the HI group). Moreover, in the sham group, the percentage (Fig. 3g) of time spent in the target quadrant (55.02 ± 12.90 vs. 24.78 ± 11.13%, *p* < 0.001) and the frequency (4.56 ± 1.32 vs. 0.75 ± 0.77, *p* < 0.001) of crossing the target platform (where the platform was previously located) were significantly higher than in the HI group (Fig. 3h). These results indicated that the spatial learning and memory function of HI group rats had been severely weakened as a result of the injury. NGR1 showed neuroprotective effects by significantly decreasing the rats’ latencies(33.43 ± 13.23, 20.57 ± 9.90, 20.78 ± 8.78, and 27.44 ± 11.43 s, respectively; *p* < 0.05 vs. the HI group) and increasing the percentage of time spent in the target quadrant (36.51 ± 13.49%, *p* < 0.01 vs. the HI group) and the frequency of crossing the target platform (1.72 ± 1.09, *p* < 0.01 vs. the HI group). However, the protective effects could be reversed by ICI 182780. The latencies of the HI + NGR1 + ICI 182780 group (44.46 ± 13.33, 33.78 ± 15.45, 34.54 ± 11.54, and 35.54 ± 15.31 s, respectively) were significantly higher than those of the NGR1 treatment group (*p* < 0.05). The same results were found in the percentage of time spent in the target quadrant (27.88 ± 9.61%, *p* < 0.05 vs. the HI + NGR1 group) and the frequency (1.03 ± 1.11, *p* < 0.01 vs. the HI + NGR1 group) of crossing the target platform. The results suggested that NGR1 might exert its protective effects by targeting ERs.

### NGR1 Increased Activity of the PI3K-Akt-mTOR Signal Pathway via Estrogen Receptors

#### In Vitro and In Vivo

**Fig. 4:**
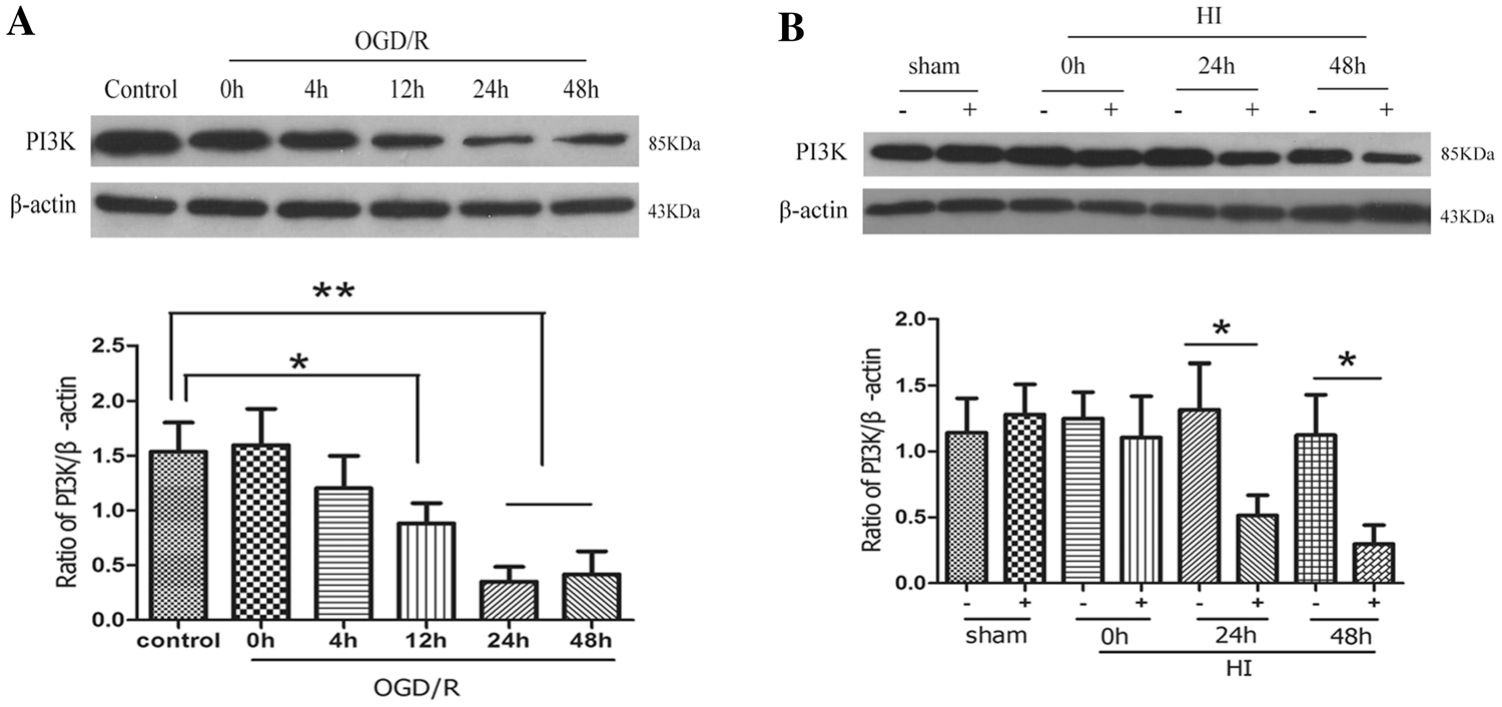
Expression of PI3K during OGD/R and HIBD. Representative Western blots for PI3K in primary cortical neurons and in HI rats. **a** PI3K was expressed at low levels 12, 24, 48 h after OGD/R. **b** Compared with the contralateral hemisphere, PI3K was expressed at low levels in the ipsilateral hemisphere 24 and 48 h after HI. (*p < 0.05; **p < 0.01 compared with control/sham groups, n = 5, mean ± SEM)

PI3K is an intracellular phosphatidylinositol kinase that plays a major role in cerebral hypoxic–ischemic injury by regulating its downstream signaling pathway. Western blot analysis was used to detect expression levels of PI3K at different times after hypoxic–ischemic injury in vitro (primary cortical neurons) and in vivo (ipsilateral hemisphere). As shown in Fig. 4a, expression of PI3K (1.54 ± 0.60 in the control group) was significantly decreased at 12 (0.88 ± 0.42, *p* < 0.05 vs. the control group), 24(0.35 ± 0.31, *p* < 0.01 vs. the control group), and 48 h (0.42 ± 0.47, *p* < 0.01 vs. the control group) of reoxygenation cortical neurons. In vivo, expression of PI3K in the ipsilateral hemisphere was significantly decreased at 24 (0.51 ± 0.34 vs. 1.32 ± 0.78, *p* < 0.05) and 48 h (0.30 ± 0.32 vs. 1.12 ± 0.69, *p* < 0.05) post HI compared with the contralateral hemisphere (Fig. 4b).

Akt is an important downstream target in the PI3K signal transduction pathway which can promote cell survival, inhibit apoptosis and maintain normal function as a key information molecule. As one of the important substrates for Akt, mTOR plays an important role in cell survival and differentiation. Among its downstream target proteins, 4EBP1 and p70S6k are the key signaling molecules, involved in cell-cycle regulation and promoting the synthesis of ribosomal translation regulatory proteins.

**Fig. 5:**
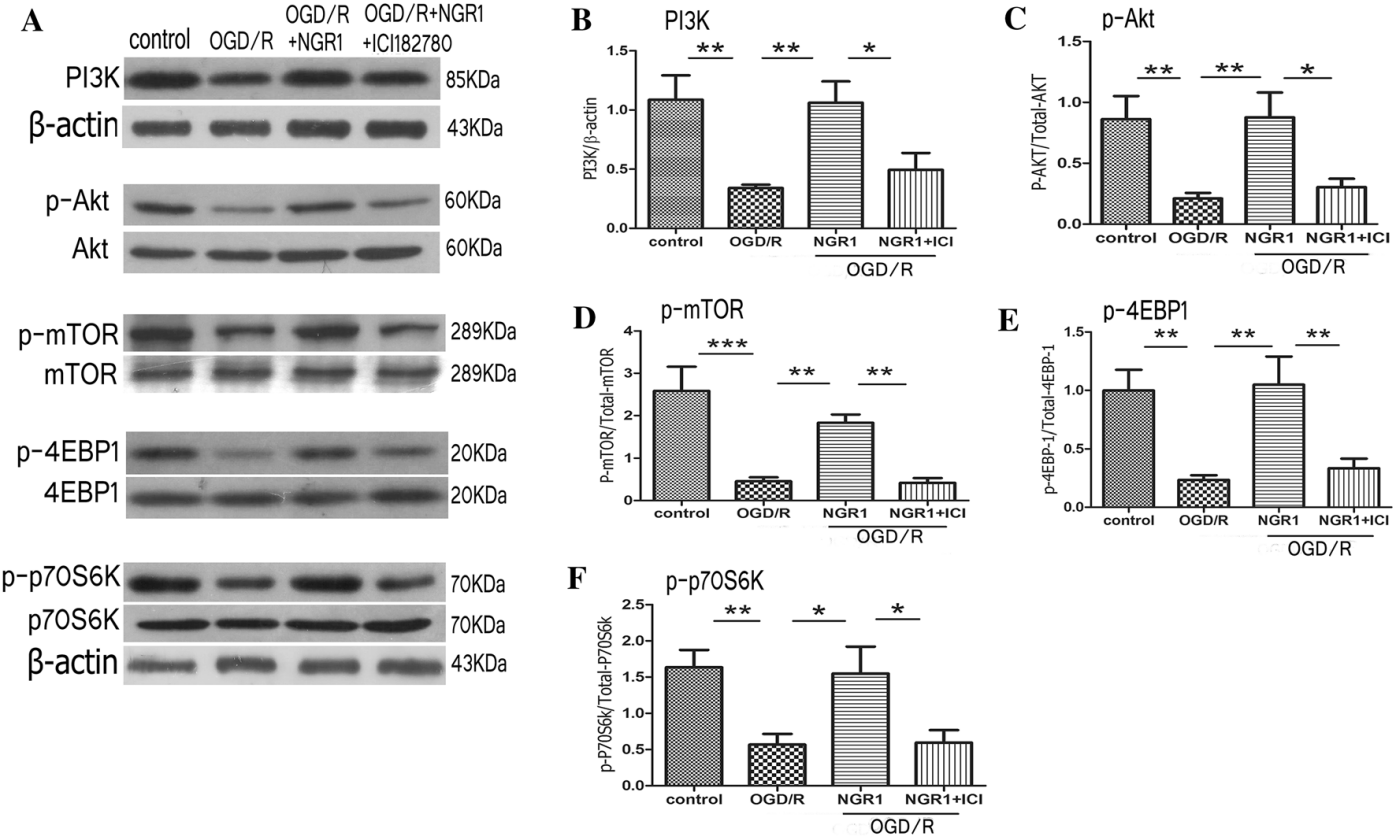
Effects of NGR1 and ICI 182780 on PI3K-Akt-mTOR-4EBP-1/p70S6K expression 24 h after OGD/R. Representative Western blots **a** for PI3K, phospho-Akt/Akt, phospho-mTOR/mTOR, phospho-p70S6K/p70S6K, and phospho-4EBP-1/4EBP-1 in primary cortical neurons. Western blot results showed that the expression of PI3K (**b**), phospho-Akt (**c**), phospho-mTOR (**d**), phospho-p70S6K (**e**), and phospho-4EBP1 (**f**) was reduced in the OGD/R group compared with the control group. NGR1 (10 mmol/l) enhanced the expression of PI3K phospho-Akt, phospho-mTOR, phospho-p70S6K, and phospho-4EBP1 in vitro. Pretreatment with ICI 182780 before NGR1 treatment could block the promoting effect. *p < 0.05; **p < 0.01; ***p < 0.001; n = 5, mean ± SEM

**Fig. 6:**
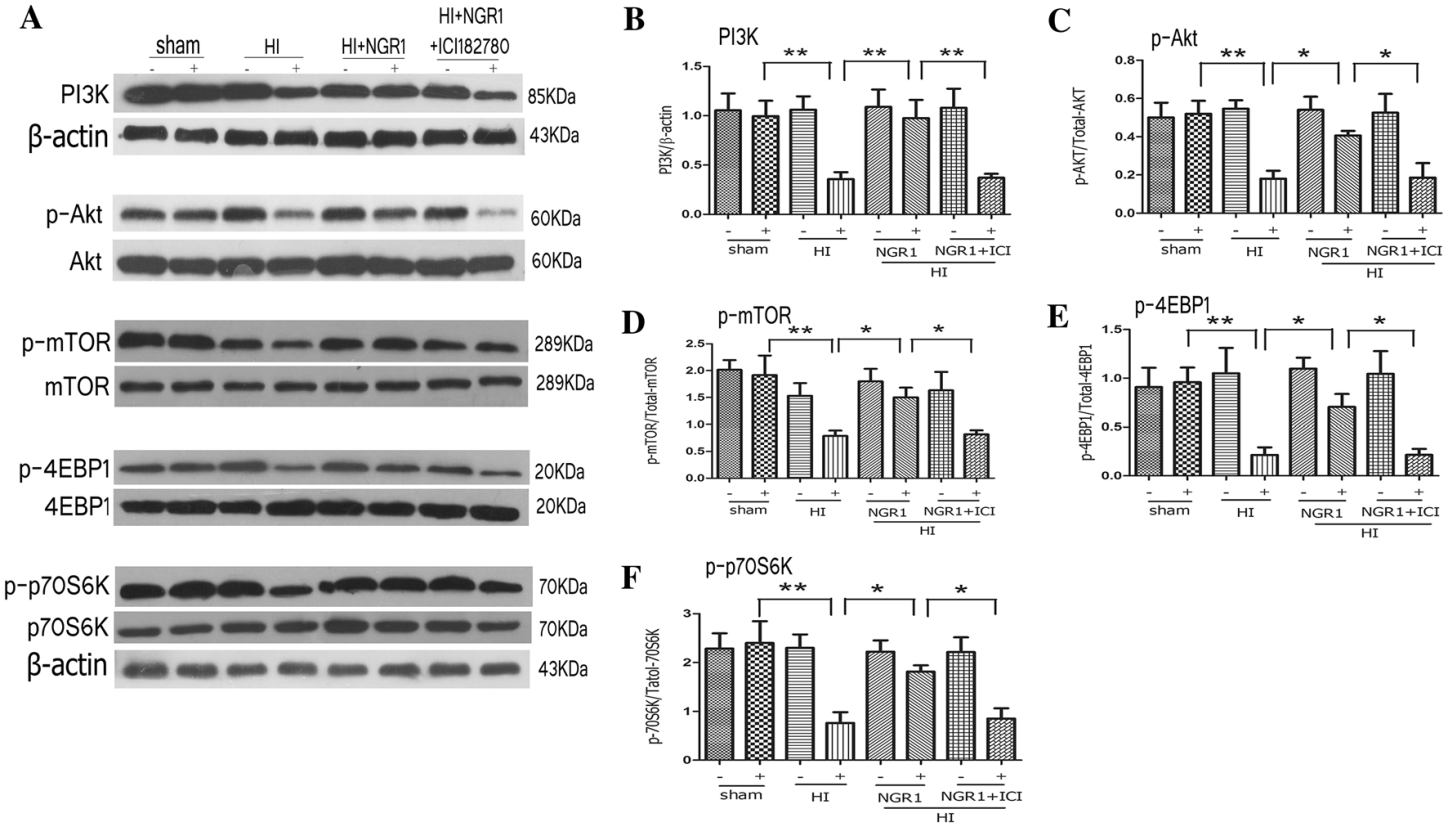
Effects of NGR1 and ICI 182780 on PI3K-Akt-mTOR-4EBP-1/P70S6K expression 24 h after HI. Representative Western blots **a** for PI3K, phospho-Akt/Akt, phospho-mTOR/mTOR, phospho-P70S6K/P70S6K, and phospho-4EBP-1/4EBP-1 in vivo. Western blot results showed that the expression of PI3K (**b**), phospho-Akt (**c**), phospho-mTOR (**d**), phospho-p70S6K (**e**), and phospho-4EBP1 (**f**) was significantly decreased in the HI group compared with the sham group. NGR1 (15 mg/kg) enhanced the expression of PI3K phospho-Akt, phospho-mTOR, phospho-p70S6K, and phospho-4EBP1 in vivo. Pretreatment with ICI 182780 before NGR1 treatment could block the promoting effects. *p < 0.05; **p < 0.01; n = 5, mean ± SEM

To detect activity of PI3K/Akt/mTOR signal path, primary neurons or hemisphere tissue were harvested at 24 h after OGD/R or HI injury for western blots. As seen in Fig. 5, the OGD/R group showed significant decrease in PI3K (0.34 ± 0.07 vs. 1.09 ± 0.46 *p* < 0.01), phospho-Akt (0.21 ± 0.10 vs. 0.86 ± 0.42, *p* < 0.01), phospho-mTOR (0.46 ± 0.21 vs. 2.58 ± 1.28, *p* < 0.001), phospho-4EBP1 (0.24 ± 0.09 vs. 1.00 ± 0.40, *p* < 0.01), and phospho-p70S6k (0.57 ± 0.33 vs. 1.63 ± 0.53, *p* < 0.01) compared with the control group. Treatment with NGR1 (10 µmol/l) increased the expression levels of PI3K (1.06 ± 0.40, *p* < 0.01 vs. the OGD/R group), phospho-Akt (0.88 ± 0.46, *p* < 0.01 vs. the OGD/R group), phospho-mTOR (1.83 ± 0.43, *p* < 0.01 vs. the OGD/R group), phospho-4EBP1 (1.05 ± 0.54, *p* < 0.01 vs. the OGD/R group), and phospho-p70S6k (1.55 ± 0.83, *p* < 0.05 vs. the OGD/R group). However, pretreatment with ICI 182780 before NGR1 treatment in vitro resulted in the down-regulation of PI3K (0.49 ± 0.32, *p* < 0.05 vs. the OGD/R + NGR1 group), phospho-Akt (0.30 ± 0.15, *p* < 0.05 vs. the OGD/R + NGR1 group), phospho-mTOR (0.42 ± 0.25, *p* < 0.01 vs. the OGD/R + NGR1 group), phospho-4EBP1 (0.33 ± 0.18, *p* < 0.01 vs. the OGD/R + NGR1 group), and phospho-p70S6k (0.60 ± 0.39, *p* < 0.05 vs. the OGD/R + NGR1 group) protein expression compared with the OGD + NGR1 group. As shown in Fig. 6, there was significant decrease in PI3K (0.36 ± 0.16 vs. 1.00 ± 0.35, *p* < 0.01), phospho-Akt (0.18 ± 0.09 vs. 0.52 ± 0.15, *p* < 0.01), phospho-mTOR (0.79 ± 0.22 vs. 1.92 ± 0.82, *p* < 0.01), phospho-4EBP1 (0.21 ± 0.18 vs. 0.96 ± 0.34, *p* < 0.01), and phospho-p70S6k (0.76 ± 0.49 vs. 2.40 ± 1.00, *p* < 0.01) compared with the sham group. Treatment with NGR1 (15 mg/kg) increased the expression levels of PI3K (0.98 ± 0.42, *p* < 0.01 vs. the HI group), phospho-Akt (0.41 ± 0.05, *p* < 0.05 vs.the HI group), phospho-mTOR (1.5 ± 0.41, *p* < 0.05 vs. the HI group), phospho-4EBP1 (0.70 ± 0.30, *p* < 0.05 vs. the HI group), and phospho-p70S6k (1.81 ± 0.29, *p* < 0.05 vs. the HI group). However, pretreatment with ICI 182780 before NGR1 treatment in vivo resulted in the down-regulation of PI3K (0.37 ± 0.09, *p* < 0.01 vs. the HI + NGR1 group), phospho-Akt (0.19 ± 0.17, *p* < 0.05 vs. the HI + NGR1 group), phospho-mTOR (0.82 ± 0.16, *p* < 0.05 vs.the HI + NGR1 group), phospho-4EBP1 (0.22 ± 0.14, *p* < 0.05 vs. the HI + NGR1 group), and phospho-p70S6k (0.85 ± 0.47, *p* < 0.05 vs. the HI + NGR1 group) protein expression compared with the HI + NGR1 group. The results indicated that NGR1 might regulate the PI3K-Akt-mTOR signal pathway via ERs in hypoxic–ischemic brain injury.

### NGR1 Downregulated JNK Signal Pathway via Estrogen Receptors in Vitro and in Vivo

**Fig. 7:**
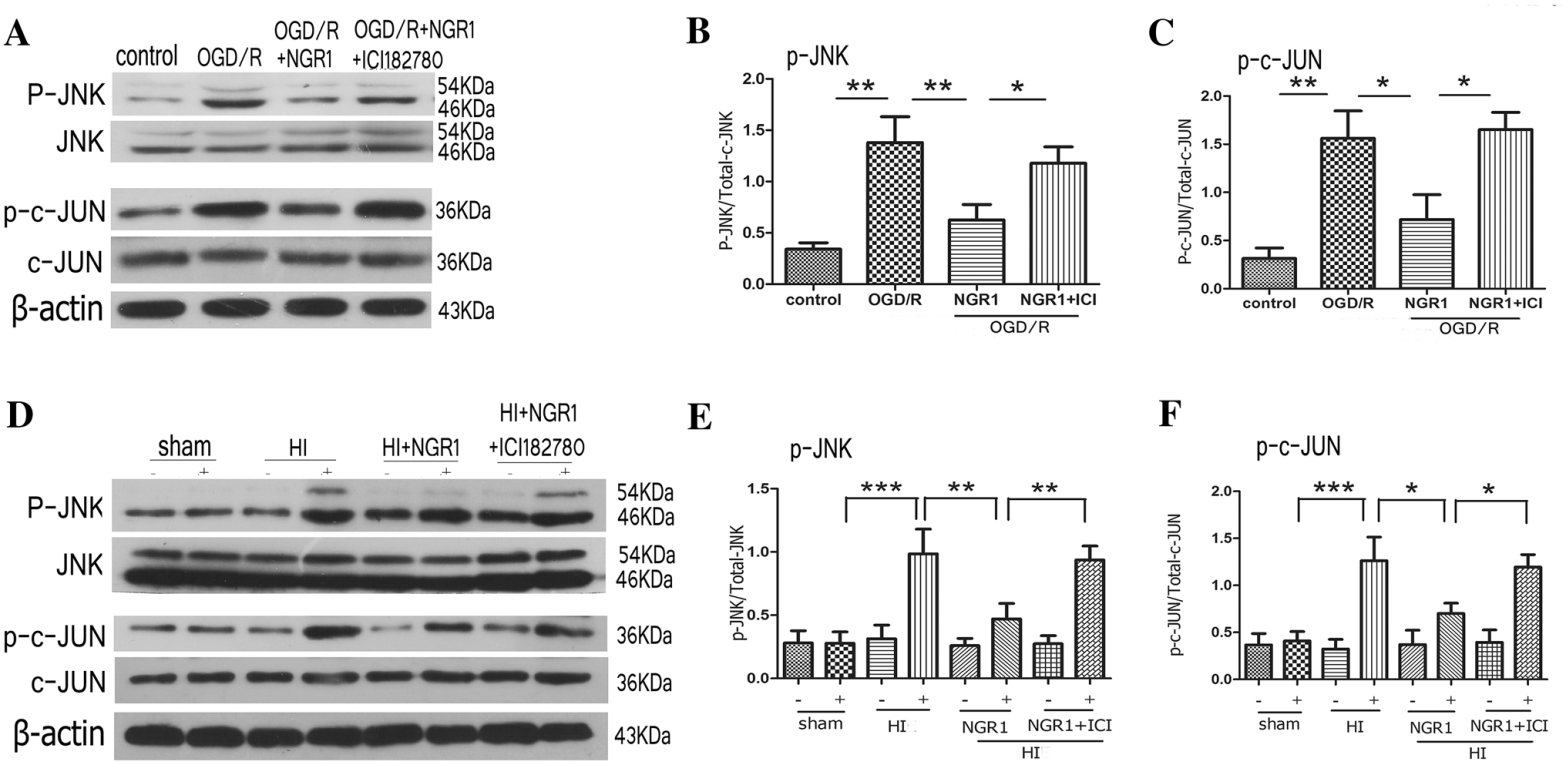
Effects of NGR1 and ICI 182780 on JNK-c-JUN expression 24 h after OGD/R and HI. Representative Western blots for phospho-JNK/JNK and phospho-c-JUN/c-JUN in primary cortical neurons (**a**) and for phospho-JNK/JNK and phospho-c-JUN/c-JUN in vivo (**d**). Western blot analysis showed that compared with the OGD/R or HI group, NGR1 inhibited the expression of phospho-JNK and phospho-c-JUN in vitro (**b, c**) and in vivo (**e, f**). Pretreatment with ICI 182780 before NGR1 treatment could block the inhibiting effects of NGR1. *p < 0.05; **p < 0.01; n = 5, mean ± SEM

The phosphorylation of JNK and c-JUN were examined 24 h after OGD/R or HI injury. Western blot analysis showed that OGD/R injury resulted in remarkably increased expression of both phospho-JNK (1.38 ± 0.56 vs. 0.34 ± 0.14, *p* < 0.01) and phospho-c-JUN (1.56 ± 0.63 vs. 0.31 ± 0.24, *p* < 0.01) in primary cortical neurons compared with the control group. NGR1 treatment significantly decreased the expression levels of phospho-JNK(0.63 ± 0.33, *p* < 0.01 vs. the OGD/R group) and phospho-c-JUN(0.72 ± 0.57, *p* < 0.05 vs. the OGD/R group), and the effects of NGR1 were blocked by ICI 182780. Pretreatment with ICI 182780 before NGR1 treatment led to higher levels of phospho-JNK (1.18 ± 0.36) and phospho-c-JUN (1.65 ± 0.40) than those in OGD/R + NGR1 group (*p* < 0.05) (Fig. 7a–c).

Similarly, in the HI group, the expression of phospho-JNK (0.99 ± 0.44 vs. 0.28 ± 0.20, *p* < 0.001) and phospho-c-JUN (1.26 ± 0.56 vs. 0.41 ± 0.22, *p* < 0.001) increased in the ipsilateral hemisphere compared with the sham group, and NGR1 attenuated the activation of phospho-JNK (0.47 ± 0.28, *p* < 0.01 vs. the HI group) and phospho-c-Jun (0.70 ± 0.24, *p* < 0.05 vs. the HI group). Pretreatment with ICI 182780 before NGR1 treatment led to higher levels of phospho-JNK (0.94 ± 0.25, *p* < 0.01 vs. the HI + NGR1 group) and phospho-c-JUN (1.19 ± 0.30 *p* < 0.05 vs. the HI + NGR1 group) than those in the NGR1 group (Fig. 7d–f).

The results indicated that NGR1 might inhibit the activity of JNK/c-JUN signal pathway by acting ERs and reduced the neuronal apoptosis.

### NGR1 Exerted Neuroprotective Effects via Estrogen Receptors and PI3K

The preceding results showed that NGR1 could exert neuroprotective effects by regulating the PI3K-Akt-mTOR/JNK signal pathways, but these effects could be reversed by blocking the ERs. Previous research [60–64] showed that PI3K could interact with ERs. To further explore the relationship between NGR1, PI3K and ERs, LY294002 (PI3K inhibitor) and 740Y-P (PI3K agonist) were used.

**Fig. 8:**
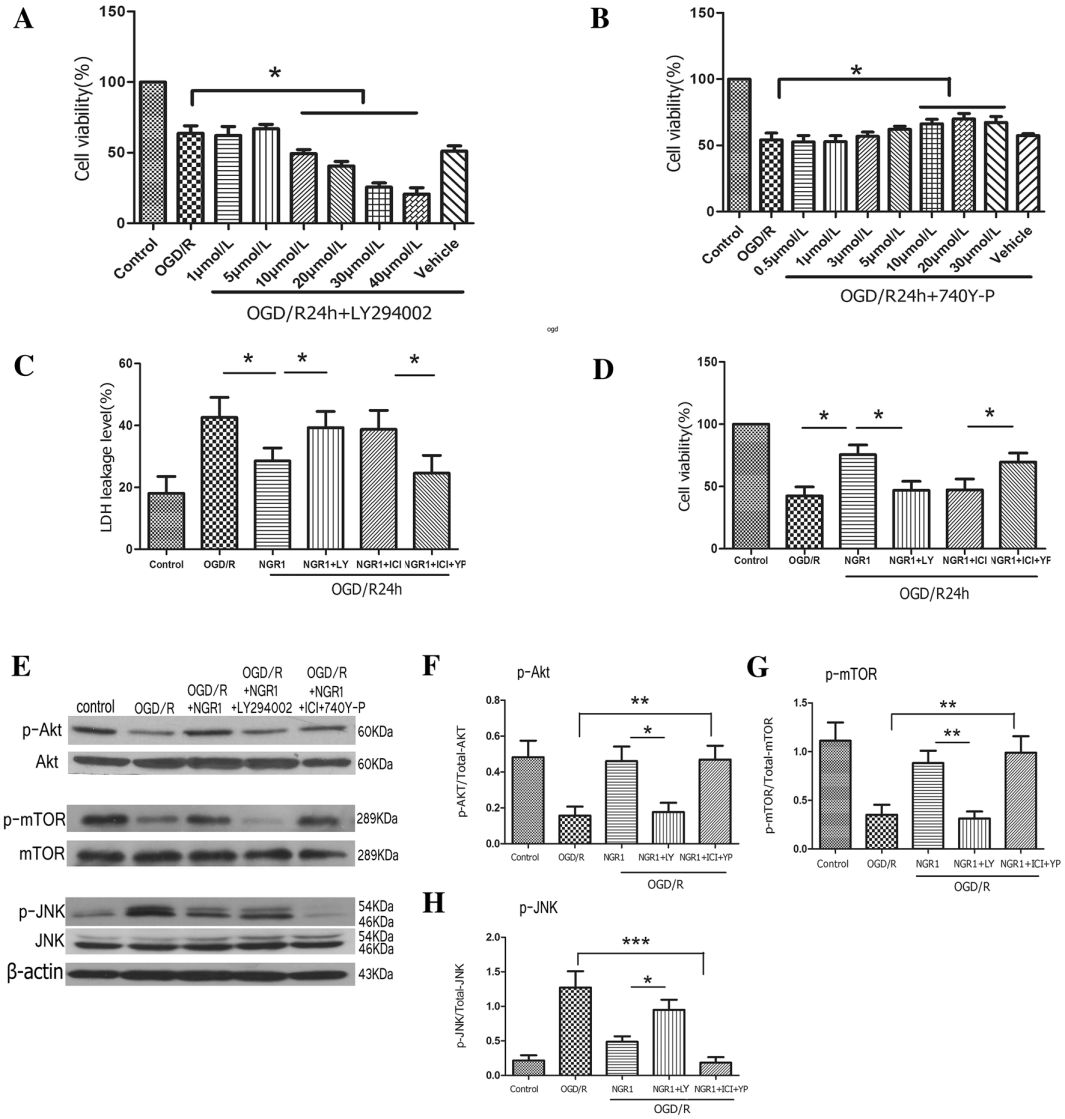
Effects of LY294002/740Y-P during OGD/R. **a** The optimal concentration of LY294002 was 20 µmol/l. **b** The optimal concentration of 740Y-P was 20 µmol/l. LY294002 treatment could accelerate LDH leakage **c** and reduce cell viability **d** in the OGD/R + NGR1 + LY294002 group compared to the NGR1 treatment group. 740Y-P treatment could promote cell viability and inhibit LDH leakage in the OGD/R + NGR1 + ICI 182780 + 740Y-P group compared to the OGD/R + NGR1 + ICI 182780 group. (*p < 0.05, n = 6, mean ± SEM). Representative Western blots **e** for phospho-Akt/Akt (**f**), phospho-mTOR/mTOR (**g**) and phospho-JNK/JNK (**h**) in primary cortical neurons. In the OGD/R + NGR1 + LY294002 group, the phosphorylation of Akt and mTOR was lower than that in the OGD/R + NGR1 group, with a higher phosphorylation of JNK than that in the OGD/R + NGR1 group; In the OGD/R + NGR1 + ICI 182780 + 740Y-P group, Akt/mTOR phosphorylation was higher and JNK phosphorylation was lower than that in the OGD/R group. *p < 0.05; ** *p* < 0.01; ***p < 0.001; n = 5, mean ± SEM

As shown in Fig. 8, with a optimum concentration of LY294002 treatment (20 µmol/l) [40] (Fig. 8a), the OGD + NGR1 + LY294002 group showed lower cell viability (46.99 ± 17.50 vs. 75.53 ± 18.94%, *p* < 0.05) and more LDH leakage (39.40 ± 7.40 vs. 28.18 ± 6.40%, *p* < 0.05) than the NGR1 treatment group, which suggested that the neuroprotective effects of NGR1 were inhibited (Fig. 8c, d). At the same time, the phosphorylation of Akt (0.18 ± 0.12 vs. 0.46 ± 0.18, *p* < 0.05) and mTOR (0.31 ± 0.16 vs. 0.88 ± 0.28, *p* < 0.01) was lower in the OGD/R + NGR1 + LY294002 group than that in the OGD/R + NGR1 group, while the phosphorylation of JNK (0.96 ± 0.32 vs. 0.49 ± 0.17, *p* < 0.05) was higher than that in the OGD/R + NGR1 group (Fig. 8e–h). To further explore the role of ERs in the PI3K signal pathway, the optimal concentration of 740Y-P was tested and found to be 20 µmol/l (Fig. 8b); this concentration was used in the following investigation. The results showed that ICI 182780 could reverse the neuroprotective effects of NGR1 and aggravate neural injury. However, when 740Y-P was used in the OGD/R + NGR1 + ICI 182780 group, the expression of phospho-Akt (0.46 ± 0.17 vs. 0.16 ± 0.11, *p* < 0.01) and phospho-mTOR (0.99 ± 0.39 vs. 0.35 ± 0.23, *p* < 0.01) was activated and the expression of phospho-JNK (0.18 ± 0.17 vs. 1.28 ± 0.50, *p* < 0.001) was inhibited compared with the OGD/R group (Fig. 8e–h). Simultaneously, the results showed higher cell viability (69.70 ± 17.52 vs. 47.34 ± 21.36%, *p* < 0.05) and less LDH leakage (24.27 ± 9.30 vs. 38.97 ± 10.20%, *p* < 0.05) in the OGD/R + NGR1 + ICI 182780 + 740Y-P group compared with the OGD/R + NGR1 + ICI 182780 group(Fig. 8c, d). These results indicated that ERs might regulate the activation of Akt-mTOR/JNK through interaction with PI3K, and NGR1 might cause PI3K activation to decrease cell damage after OGD/R by targeting ERs.

## Discussion

HIE is a common neurologic disease in newborns, but there is currently a lack of promising therapy [3]. Many studies have shown that estrogen provides neuroprotective effects in experimental cerebral ischemia [20, 21]. These protective effects are mediated by ligand interactions with two primary classical ERs, ERα and ERβ [65]. Research has shown that the distribution patterns of ERα and ERβ are similar in male and female brains. Especially in the cortical and hippocampal regions [66], sex differences were found to be absent [67]. However, studies suggested that estrogen exhibited universal protection against experimental ischemia injury via ERs in female but not male brains [68]. The differences may be due at least in part to the fact that circulating estrogens have free access to all brain regions. As a phytoestrogen, NGR1 has been found to exhibit a number of treatment effects and exert direct anti-inflammatory and anti-apoptotic effects on cardiomyocytes [26], vascular endothelial cells [69], podocytes [70], and neurons [18, 44] through acting ERs. Some scholars reported that NGR1 treatment significantly improved cognitive function in the APP/PS1 double-transgenic mouse model of Alzheimer’s disease [71]. One study demonstrated neuroprotective effects of NGR1 in an adult rat model of cerebral ischemia/reperfusion [18]. However, research has revealed that the immature brain responded differently to treatment than the mature brain in laboratory animals [3]. In fact, therapies designed to ameliorate brain injury in adults may worsen outcomes in neonates [72]. Hence, effective therapies for neonatal HIE need to be explored. Although some preliminary experimental results are available [19], whether NGR1 exerts short-term or long-term protective effects and the underlying mechanisms are largely unknown. Therefore, the evaluation of the early effects and long-term therapeutic effects of NGR1 is of great clinical significance.

In the present study, a series of experiments were designed to explore the neuroprotective effects and underlying mechanisms of NGR1 in a neonatal hypoxic-ischemic injury model. The pivotal findings are as follows. (1) NGR1 significantly attenuated neuronal injury in the neonatal HI model in vitro and in vivo. Most importantly, NGR1 had contributed to the long-term recovery of neurological function in the HI rats. (2) NGR1 exerted neuroprotective effects through regulating the PI3K-Akt-mTOR/JNK signal pathways by targeting ERs.

HIE [11] can develop as a result of circulatory and energy metabolism disorders, leading to a series of pathophysiological processes, including oxidative stress, mitochondrial impairment, apoptosis, and necroptosis. These injuries in the developing brain often lead to lasting neurological impairments, such as cerebral palsy, epilepsy, mental retardation, and learning and memory disorders. Therefore, reducing neuronal death and promoting neuronal survival and proliferation are important strategies for reducing the occurrence of long-term neurological sequelae [26]. Our results indicated that NGR1 possessed protective effects both in vitro and in vivo. NGR1 was observed significantly to improve neuronal cell viability and reduce the LDH leakage rate 4 and 24 h after OGD/R (Fig. 1). The inhibition of cortical neuronal apoptosis was observed 24 h after HI injury and the decrease of infarct volume was examined 48 h after HI injury in HI + NGR1 group (Fig. 2). These findings are consistent with a recent study in an adult cerebral ischemia–reperfusion brain injury model, which found that NGR1 therapy reduced brain damage after ischemia [18]. However, that study used a higher concentration of NGR1 (25 mmol/l in vitro and 20 mg/kg in vivo) than our study (10 mmol/l in vitro and 15 mg/kg in vivo). There may be two reasons for the difference. (1) We used cells from different culture days and rats of different ages. (2) NGR1 was administered after OGD/R or HI in our study, not as a pretreatment. Importantly, our results indicated that NGR1 contributed to the long-term recovery of neurological function in the neonatal HI model in addition to reducing apoptosis. NGR1 treatment reduced brain atrophy 6 weeks after HI injury (Fig. 2). Moreover, the results of beam walking (5 weeks after HIE) and the water maze test (5–6 weeks after HIE) showed that NGR1 significantly restored limb coordination and improved learning and memory in the impaired rats (Fig. 3).

Hypoxic–ischemic brain injury directly results in a large amount of neuronal death. Therefore, reducing neuronal death and promoting neuronal survival and proliferation are important strategies for reducing the occurrence of long-term neurological sequelae [26]. Apoptosis is reported to be responsible for a significant proportion of the HI-induced neuronal loss [72], and multiple apoptosis-related signal pathways, such as PI3K-Akt-mTOR/JNK, are involved in neuronal death after stroke [34, 40, 41]. Our results showed significant inhibition of the PI3K-Akt-mTOR-4EBP1/p70S6k signal pathway at 24 h following OGD/R or HI injury (Figs. 5, 6). At the same time, JNK—another important signaling protein downstream of Akt, which can be inhibited by Akt directly or indirectly—was significantly activated. These results suggested that neuronal apoptosis might be related to the inhibition of PI3K-Akt-mTOR and the activity of JNK-c-JUN during HIBD. Some other researchers [44, 49–51, 73, 74] have found similar results indicating that cerebral ischemia induced the robust activation of JNK signaling and inhibition of PI3K-Akt-mTOR pathway activity. NGR1 treatment could increase the expression of PI3K, phospho-Akt, and phospho-mTOR (Figs. 5, 6) and reduce the activity of the JNK signaling pathway 24 h after OGD/R or HI brain injury (Fig. 7). These results indicated that NGR1 could likely reduce neuronal apoptosis by regulating the activity of the PI3K-Akt-mTOR/JNK signal pathways. NGR1 treatment could improve the cell survival rate in vitro and reduce infarct volume and promote long-term neurobehavioral recovery and improvement in vivo by inhibiting neuronal apoptosis. Previous studies showed that mTOR accelerated angiogenesis [75] and neuronal regeneration [76] in many neurologic injuries in addition to reducing neuronal apoptosis. Perhaps the long-term protective effects of NGR1 were also related to its activation of mTOR and promotion of neuroregeneration.

We further explored whether NGR1 achieved its neuroprotective effects via ERs. As a predominant phytoestrogen extracted from *P. notoginseng*, NGR1 was previously found to perform its function through acting ERs [13, 15, 18, 19]. Mounting evidence showed that ERα and ERβ expression was reduced during neuronal ischemia [19, 77]. Kraczkowski [78] indicated that the downregulation of ERs might be related to the ontogenesis of brain µ-opioid receptors during HIBD. As an ERs agonist, NGR1 may act on ERα/β and improve the role of ERs during HIBD [18, 19]. Our results indicated that pretreatment with ICI 182780 reduced the survival rate of cortical neurons in vivo and increased brain edema and cerebral infarction volume in vitro compared with the HI + NGR1 group. Moreover, the long-term protective effects of NGR1 were suppressed by ICI 182780. These results suggested that NGR1 exerted its protective effects via ERs.

Studies on a variety of cells—such as endothelial cells [79], MCF-7 breast cancer cells [80], and neurons [81–83]—have found that ERs can interact directly with PI3K or bind to the PI3K p85 subunit through scaffold proteins such as CAV-1, connective proteins such as Src and Shc, and growth factors, then activate the downstream Akt, causing a series of signal pathway cascades, such as the Akt-mTOR/JNK signal pathway [60–63]. Our results showed that pretreatment with ICI 182780 could inhibit the activity of PI3K-Akt-mTOR and increase the activity of the JNK signal pathway. These results suggested that NGR1 regulated the PI3K-Akt-mTOR/JNK signal pathways via acting ERs. In order to further validate this finding, we used LY294002 (PI3K inhibitor) and 740Y-P (PI3K agonist) to perform related experiments. The results (Fig. 8) revealed that the protective effects of NGR1 were significantly inhibited after adding LY294002, the expression of phospho-Akt and phospho-mTOR decreased and that of JNK increased in the OGD/R + NGR1 + LY294002 group. However, 740Y-P could reverse the inhibition of NGR1’s neuroprotective effects induced by ICI182780. Simultaneously, phospho-Akt expression increased and phospho-JNK expression decreased in the 740Y-P agonist group. These results suggested that NGR1 might exert a neuroprotective effects by targeting ERs and regulating PI3K.

In conclusion, the present study demonstrated that NGR1 inhibited neuronal apoptosis and promoted neuronal survival, exerting an important neuroprotective effects against HIBD in neonates through targeting ERs and regulating the PI3K-Akt-mTOR/JNK signal pathway. Our findings suggested that NGR1 might be a potent new therapeutic compound for neonatal hypoxia–ischemia brain damage treatment.
